# A Study of the Significance of Platelet Indices in Patients With Preeclampsia Attending a Tertiary Care Center in Jharkhand

**DOI:** 10.7759/cureus.104658

**Published:** 2026-03-04

**Authors:** Deepanshu Singh, Manoj K Paswan, Sunil Kumar Mahto, Shipha Akanchha Kujur, Deepali Tirkey

**Affiliations:** 1 Pathology, Rajendra Institute of Medical Sciences, Ranchi, IND

**Keywords:** eclampsia, mean platelet volume (mpv), platelet count (pc), plateletcrite (pct), platelet distribution width (pdw), pre-eclampsia, pregnancy-induced hypertension (pih)

## Abstract

Introduction

Preeclampsia is a multisystem hypertensive disorder of pregnancy that remains a major cause of maternal and perinatal morbidity and mortality worldwide. It arises after 20 weeks of gestation and is characterized by new-onset hypertension with proteinuria or end-organ dysfunction. Abnormal placentation and placental ischemia trigger the release of anti-angiogenic factors such as soluble FMS-like tyrosine kinase-1 (sFLT-1) and soluble endoglin, leading to endothelial dysfunction and platelet activation. Consequently, platelet indices, namely, platelet count (PC), mean platelet volume (MPV), platelet distribution width (PDW), and plateletcrit (PCT), may serve as accessible markers of disease severity.

Methodology

This 18-month observational cross-sectional study was conducted at the Department of Pathology, Rajendra Institute of Medical Sciences (RIMS). Eighty pregnant women (≥18 years, >20 weeks of gestation) with preeclampsia were included. Patients with pre-existing hypertension, chronic renal disease, diabetes mellitus, or hematological disorders were excluded, as these conditions can independently influence platelet count and platelet activation. Their exclusion ensured that changes in platelet indices could be more reliably attributed to preeclampsia rather than confounding comorbidities.

Results

Among 80 patients (mean age: 26.2 years), most were primigravida and presented near term; 58.75% progressed to eclampsia. Thrombocytopenia was observed in 23.75%, while elevated MPV and PDW were noted in 27.5% and 60%, respectively. PDW showed the strongest correlation with disease severity. Neonatal outcomes were favorable in 96.25% of cases.

Conclusions

PDW was the platelet index most strongly associated with progression to eclampsia. Platelet count showed a weaker predictive relationship, while MPV and PCT demonstrated a moderate association with increasing disease severity. Overall, platelet indices, particularly PDW, showed a statistically significant association with disease severity, supporting their potential utility in the clinical assessment and monitoring of preeclampsia.

## Introduction

Preeclampsia is a multifactorial hypertensive disorder affecting 3%-8% of pregnancies worldwide and remains a significant cause of maternal and perinatal morbidity and mortality [1,2]. It presents after 20 weeks of gestation with new-onset hypertension (≥140/90 mmHg) and proteinuria (≥300 mg/24 hour), and may be accompanied by systemic complications such as thrombocytopenia, renal and hepatic dysfunction, pulmonary edema, and neurological disturbances [3,4].

Its pathogenesis involves abnormal trophoblastic invasion and defective spiral artery remodeling, leading to placental ischemia and release of anti-angiogenic factors such as soluble FMS-like tyrosine kinase-1 (sFLT-1) and soluble endoglin. These changes result in endothelial dysfunction, oxidative stress, systemic inflammation, and subsequent platelet activation and coagulation cascade activation [5-7].

Platelet indices derived from routine complete blood counts, namely, platelet count (PC), mean platelet volume (MPV), platelet distribution width (PDW), and plateletcrit (PCT), have emerged as accessible markers of disease severity. Thrombocytopenia reflects increased platelet consumption, while elevated MPV and PDW indicate enhanced platelet activation and turnover. Reduced PCT signifies decreased total platelet mass, collectively reflecting the hypercoagulable state in preeclampsia [8-10]. These parameters are simple, cost-effective, and widely available, making them especially valuable in resource-limited settings.

The primary objective of this study was to evaluate the association between platelet indices (platelet count, MPV, PDW, and PCT) and progression from preeclampsia to eclampsia. The secondary objective was to compare the diagnostic performance of these indices using receiver operating characteristic (ROC) curve analysis to assess their potential role in clinical risk stratification. As this was a cross-sectional study, the analysis focused on evaluating statistical associations and diagnostic performance rather than prospective prediction.

## Materials and methods

Study design and setting

This 18-month observational cross-sectional study was conducted from February 2024 to August 2025 in the Department of Pathology in collaboration with the Department of Obstetrics and Gynecology at Rajendra Institute of Medical Sciences (RIMS), Ranchi, Jharkhand, India.

Study population

The study included pregnant women aged ≥18 years with gestational age >20 weeks diagnosed with preeclampsia, defined as systolic blood pressure (SBP) ≥140 mmHg and/or diastolic blood pressure (DBP) ≥90 mmHg measured on two occasions at least four hours apart, along with proteinuria (≥300 mg/24 hours, protein-creatinine ratio ≥0.3, or dipstick positivity).

Patients with pre-existing hypertension, chronic renal disease, diabetes mellitus, or known hematological disorders were excluded to minimize potential confounding factors.

A consecutive sampling method was employed, wherein all eligible patients meeting the inclusion criteria during the study period were enrolled until the required sample size was achieved.

Sample size calculation

The sample size was calculated using the formula:

n = Z²P(1−P) / d²

Assuming a prevalence (P) of 11% from previous literature [11], a 95% confidence interval (Z = 1.96), and a margin of error (d) of 7%, the estimated sample size was approximately 80 participants. Accordingly, 80 eligible participants were included in the final analysis.

Data collection and laboratory analysis

Two milliliters of venous blood were collected from each participant under aseptic precautions in ethylenediaminetetraacetic acid (EDTA)-anticoagulated vials prior to initiation of treatment.

Samples were analyzed using a Horiba Yumizen H2500 automated hematology analyzer (Horiba Medical, Kyoto, Japan). The analyzer was calibrated according to the manufacturer’s guidelines, and internal quality control procedures were performed daily to ensure accuracy and reliability of measurements.

The following platelet parameters were measured: platelet count, mean platelet volume (MPV), platelet distribution width (PDW), and plateletcrit (PCT).

Statistical analysis

Statistical analysis was performed using Jamovi version 2.5 (The Jamovi Project, Sydney, Australia) via the Jamovi Cloud platform.

Descriptive statistics were expressed as mean ± standard deviation for continuous variables and frequencies (percentages) for categorical variables. Receiver operating characteristic (ROC) curves were generated to assess the diagnostic performance of platelet indices. Sensitivity, specificity, and area under the curve (AUC) were calculated.

A p-value of <0.05 was considered statistically significant.

## Results

A total of 80 participants diagnosed with preeclampsia (BP ≥140/90 mmHg with proteinuria after 20 weeks of gestation) were included. All were aged ≥18 years, with a mean age of 26.2 years. The largest proportion belonged to the 23-27 years age group (33.75%, n=27), while the smallest were ≥38 years (2.5%, n=2). The mean gestational age at presentation was 37.3 weeks, with most cases between 36 and 40 weeks (67.5%, n=54) and the fewest between 25 and 30 weeks (3.75%, n=3). Primigravida constituted the majority (63.75%, n=51), followed by multigravida, with gravida four being the least represented (2.5%, n=2). Among primigravida patients, 30 (59%) progressed to eclampsia, indicating a higher risk in this group.

Most participants had a systolic blood pressure (SBP) of 161-180 mmHg (50%, n=40), followed by 140-160 mmHg (30%, n=24) and 181-199 mmHg (20%, n=16); none had an SBP ≥200 mmHg. A diastolic blood pressure (DBP) of 101-110 mmHg (45%, n=36) was the most common, followed by 91-100 mmHg (30%, n=24) and >110 mmHg (25%, n=20). All participants had proteinuria, most frequently 2+ (43.75%, n=35), while 4+ was seen in one case (1.25%). A prior history of pregnancy-induced hypertension (PIH) was present in 8.75% (n=7). Overall, 47 (58.75%) cases progressed to eclampsia. Fetal outcomes were favorable in 96.25% (n=77), with three (3.75%) stillbirths.

Detailed demographic, clinical, laboratory, and outcome parameters are summarized in Table 1.

**Table 1:** Demographic, Clinical, Laboratory, and Outcome Parameters PIH: pregnancy-induced hypertension

Parameter	Category	Number/percentage
Age group (years)	18-22	24 (30%)
	23-27	27 (33.75%)
	28-32	17 (21.25%)
	33-37	10 (12.5%)
	≥38	2 (2.5%)
Period of gestation (weeks)	25-30	3 (3.75%)
	31-35	17 (21.25%)
	36-40	54 (67.5%)
	≥41	6 (7.5%)
Gravida	G1	63.75%
	G2	20%
	G3	8.75%
	G4	2.5%
	G5	5%
Systolic blood pressure (mmHg)	140-150	30%
	161-180	50%
	181-200	20%
	>200	0%
Diastolic blood pressure (mmHg)	91-100	30%
	101-110	45%
	>110	25%
Urine dipstick protein	1+	31.25%
	2+	43.75%
	3+	23.75%
	4+	1.25%
History of PIH	Yes	8.75%
	No	91.25%
Progression to eclampsia	Yes	58.75%
	No	41.25%
Fetal outcome	Healthy	96.25%
	Stillbirth	3.75%

**Figure 1:**
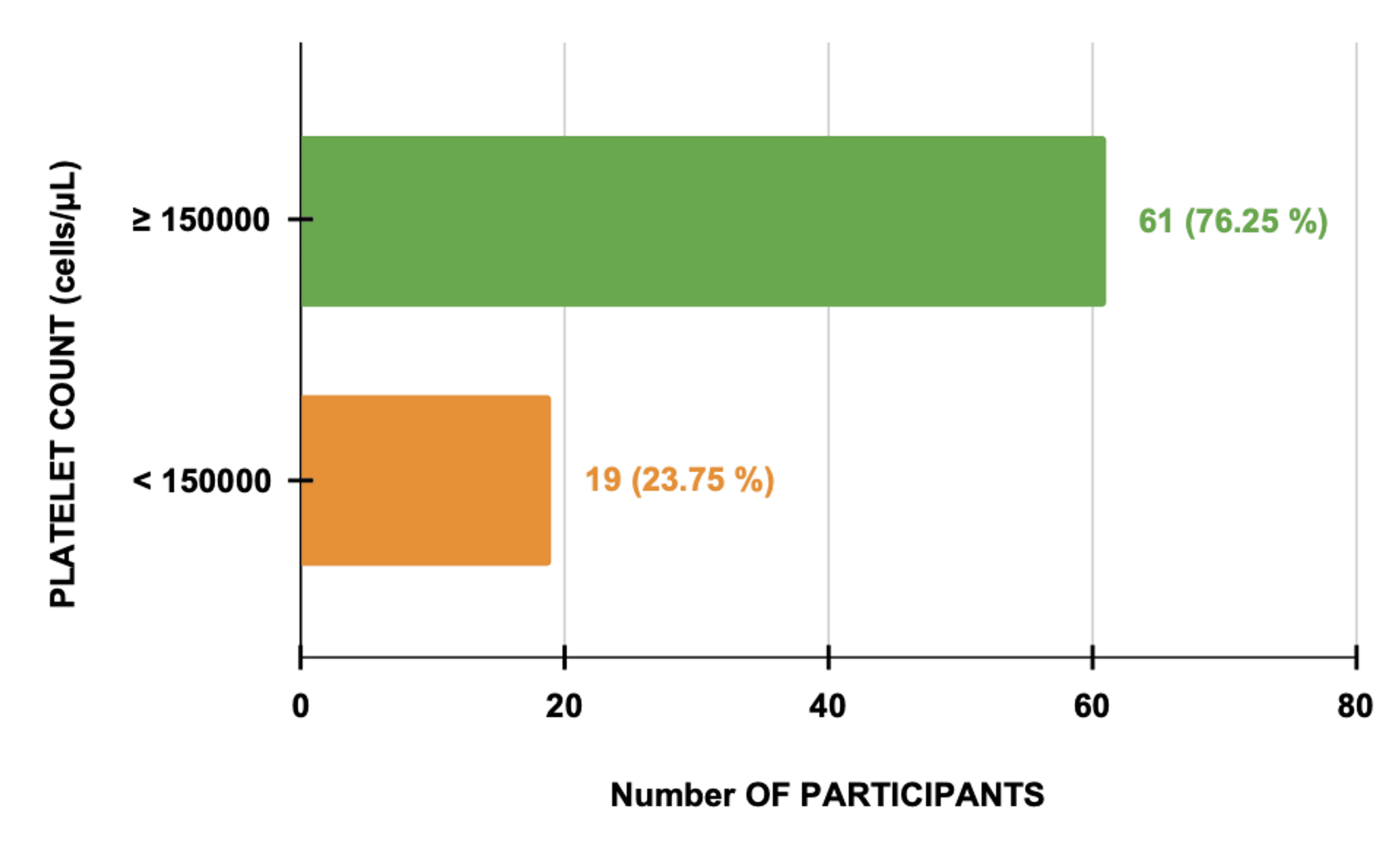
Platelet Count

Thrombocytopenia was observed in 19 (23.75%) cases, all of whom progressed to eclampsia (Figure 1). However, among participants with normal platelet counts (76.25%, n=61), 34 (55.7%) also progressed, indicating limited predictive value of platelet count alone.

**Figure 2:**
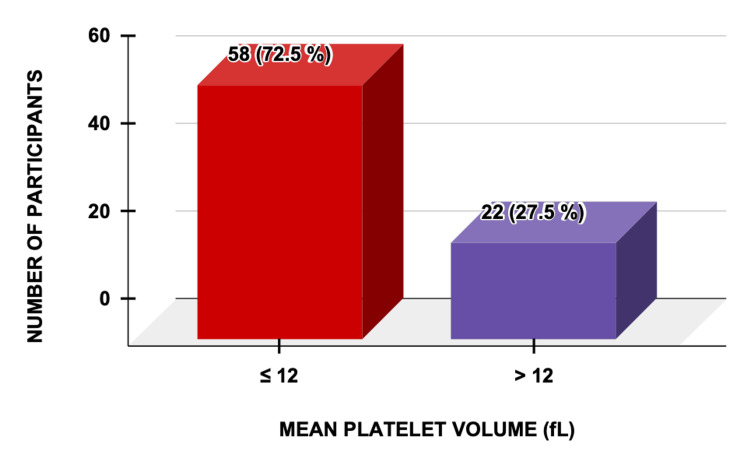
Mean Platelet Volume

Elevated mean platelet volume (MPV) was noted in 22 (27.5%) cases, predominantly in those who developed eclampsia, while 58 (72.5%) showed normal values (Figure 2).

**Figure 3:**
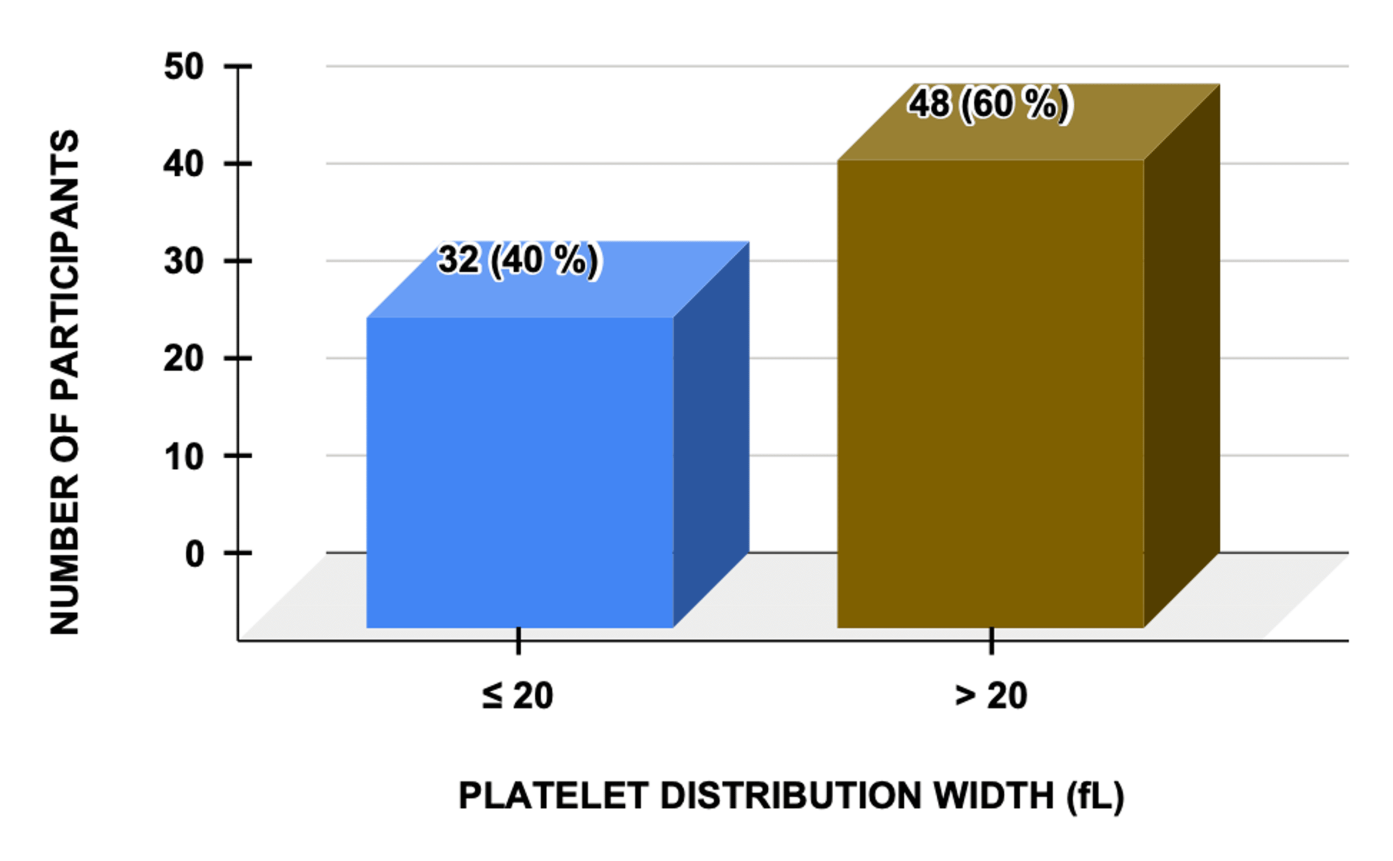
Platelet Distribution Width

Increased platelet distribution width (PDW) was observed in 48 (60%) cases, showing the strongest association with disease severity and progression (Figure 3).

**Figure 4:**
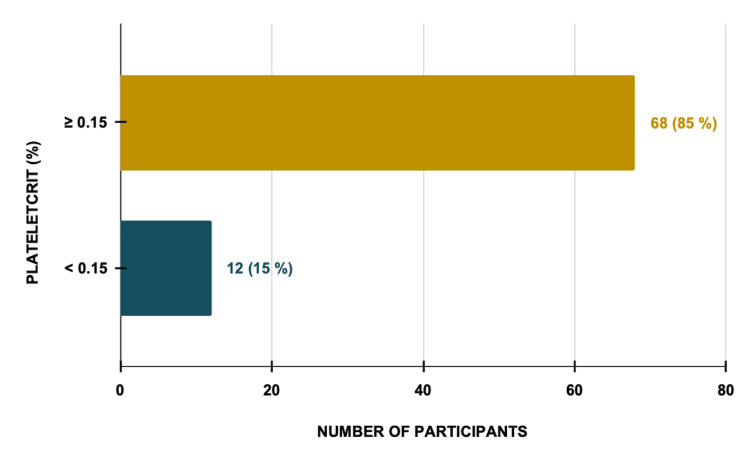
Plateletcrit

Plateletcrit (PCT) remained within normal limits in most cases (85%, n=68), with a mild decrease in 12 (15%) cases (Figure 4).

**Figure 5:**
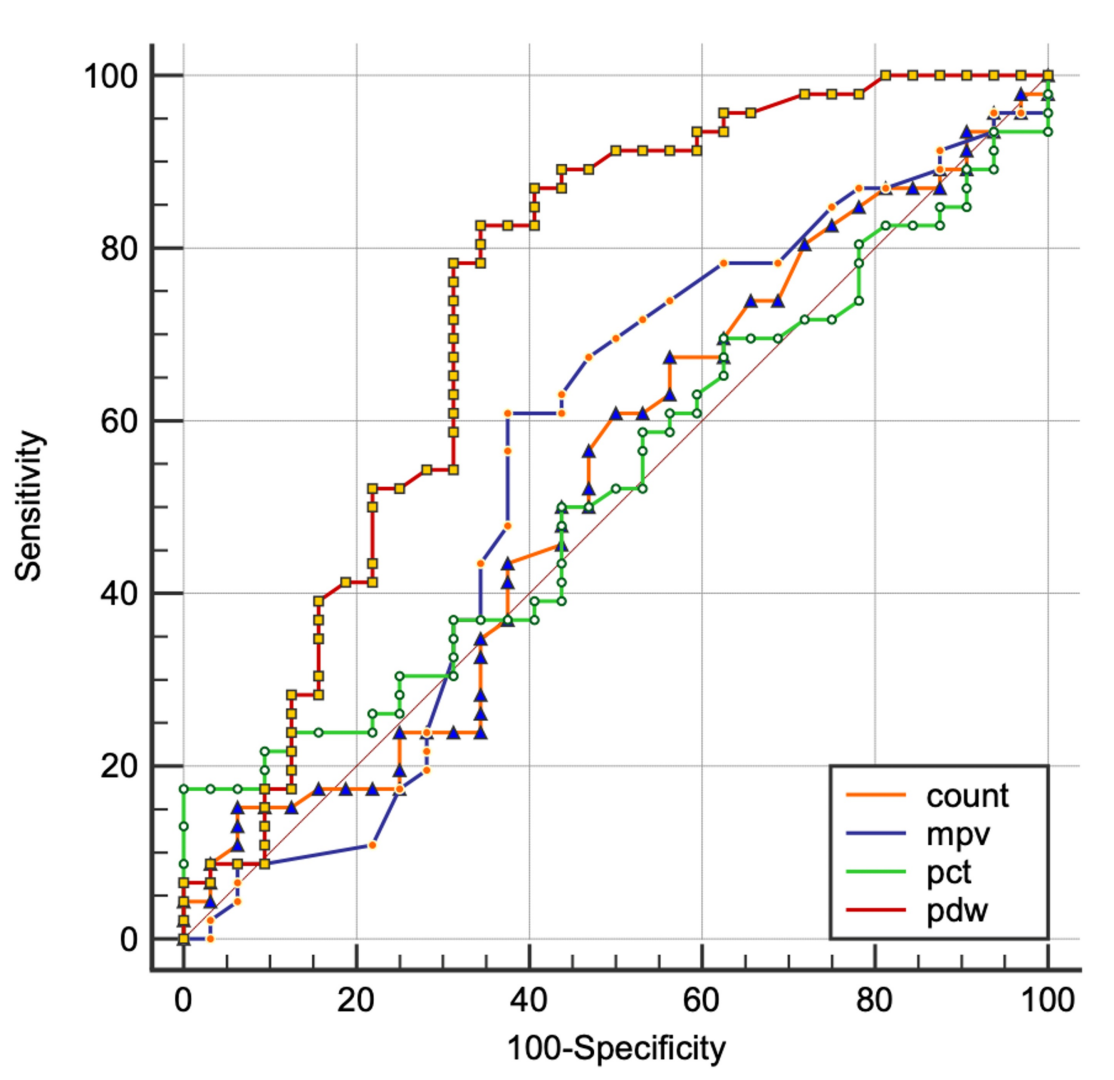
ROC Curve Analysis (Comparison of ROC Curves) count: platelet count, mpv: mean platelet volume, pct: plateletcrit, pdw: platelet distribution width, ROC: receiver operating characteristic

The ROC curve demonstrates that platelet distribution width (PDW) has the highest diagnostic accuracy for predicting progression to eclampsia, followed by mean platelet volume (MPV). Platelet count and plateletcrit (PCT) show comparatively lower predictive performance (Figure 5).

No participant developed hemolysis, elevated liver enzymes, and low platelets (HELLP) syndrome, thrombosis, or organ dysfunction, and all had normal prothrombin time/international normalized ratio (PT/INR) values, confirming inclusion at the preeclampsia stage.

## Discussion

The likelihood of developing preeclampsia was 2.68 times higher among primigravida women compared to multigravida, with a higher frequency observed in primigravida (63.75%), consistent with previous reports [12]. All participants were ≥18 years, with a mean age of 26.2 years, and the highest proportion was in the 23-27-year age group.

Thalor et al. reported that most patients had systolic blood pressure (SBP) between 140 and 149 mmHg, with a maximum of 190 mmHg [13]. In contrast, the majority of patients in our study had SBP between 161 and 180 mmHg, with the highest value reaching 200 mmHg.

A meta-analysis by Teeuw et al. reported urine dipstick protein 1+ sensitivity and specificity of 68% and 85%, respectively [14]. Our study demonstrated higher sensitivity (73.91%) but lower specificity (38.24%) at the same cutoff.

Our study demonstrated a mild reduction in platelet count in severe preeclampsia, consistent with Han et al. [15], who suggested that thrombocytopenia may reflect physiological pregnancy changes rather than preeclampsia. Similar findings were reported by Freitas et al. [16], indicating that platelet count serves as a supportive but non-definitive marker.

Dadhich et al. reported increasing MPV with advancing gestation and disease severity [17], which was also observed in our study. Elevated MPV preceding clinical diagnosis was reported by Dundar et al. [9], whereas AlSheeha et al. [8] and Altınbas et al. [18] found no significant differences. Temur et al. reported MPV sensitivity and specificity of 58.7% and 61.7%, respectively [19], compared to 65.22% and 41.18% in our study. However, Ceyhan et al. reported no prognostic value of MPV or platelet count [20], highlighting methodological variability.

Although Doğan et al. found no significant PDW differences [21], several studies reported significantly elevated PDW in preeclampsia [10]. In our study, PDW was significantly higher in patients progressing to eclampsia and correlated positively with blood pressure, suggesting enhanced platelet turnover. These findings align with those of Yang et al., who reported an AUC of 0.74 [10], comparable to our AUC of 0.747.

While previous studies reported significantly reduced PCT with good predictive value (AUC: 0.712) [22], our study showed limited utility (AUC: 0.538).

Comparative ROC analysis with Freitas et al. demonstrated comparable PDW performance (AUC: 0.747 versus 0.77), with higher sensitivity (82.98% versus 55.17%) but slightly lower specificity (66.67% versus 86.21%) [16]. MPV, platelet count, and PCT showed lower AUC values (0.551, 0.522, and 0.538, respectively) compared to Freitas et al., indicating reduced diagnostic accuracy.

Limitations

The single-center, cross-sectional design limits generalizability and prevents the establishment of causal or temporal relationships; therefore, definitive predictive inferences cannot be made. The absence of serial trimester-wise monitoring of platelet indices may have reduced predictive strength. Although major comorbid conditions were excluded, residual confounding, including demographic factors such as age and parity, and potential selection bias cannot be completely ruled out. Additionally, platelet parameters may be influenced by biological variability and pre-analytical factors, which could affect measurement precision. Larger prospective multicentric studies are warranted to validate these findings.

## Conclusions

This study assessed platelet indices in preeclampsia and their utility in predicting progression to eclampsia. Most patients presented at 36-40 weeks with severe hypertension and 2+ proteinuria, with generally favorable fetal outcomes.

Platelet count and plateletcrit had limited diagnostic value, whereas PDW and MPV showed better sensitivity and specificity. As part of routine complete blood count testing, these indices may serve as simple, cost-effective adjunctive tools for clinical risk assessment.
